# The impact of perceived organizational support on professional identity in Chinese kindergarten teachers: the mediating and moderating roles of achievement goal orientations and job stress

**DOI:** 10.3389/fpsyg.2025.1723296

**Published:** 2026-01-12

**Authors:** Jingjing Li, Xingcan Ni, Manna Wang, Zhiqing Ou, Jing Chen, Tingting Chen, Chengfu Yu

**Affiliations:** 1 School of Health Management, Guangzhou Medical University, Guangzhou, China; 2 Department of Psychology, Research Center of Adolescent Psychology and Behavior, School of Education, Guangzhou University, Guangzhou, China; 3 Music Department, Secondary Vocational Department, Guangzhou Preschool Teachers College, Guangzhou, China

**Keywords:** achievement goal orientations, job stress, kindergarten teachers, perceived organizational support, professional identity

## Abstract

**Introduction:**

Kindergarten teachers often experience significant job stress and have increasingly high turnover rates. Professional identity is associated with burnout and turnover intention, underscoring the importance of exploring the key factors that enhance it in teachers. Professional identity development is influenced by the working environment and constrained by teachers’ individual factors, such as achievement goal orientations and job stress. However, limited number of studies have investigated the mediating role of achievement goal orientation in the relationship between organizational support and professional identity. Therefore, this study focused on the impact of perceived organizational support on kindergarten teachers’ professional identities and examined the mediating role of their achievement goals and moderating role of job stress.

**Methods:**

This study conducted a questionnaire survey of 1,475 kindergarten teachers in China through random sampling (97% women, *M_age_* = 34.01 years, SD = 8.41 years), measuring perceived organizational support, job stress, achievement goal orientation, and professional identity.

**Results:**

The results indicated that achievement goal orientations mediated the relationship between perceived organizational support and professional identity. Additionally, job stress moderated the influence of perceived organizational support on specific goal orientations.

**Discussion:**

This study makes unique contributions by unraveling the distinct motivational pathways through which perceived organizational support influences professional identity, while highlighting the contextual role of job stress in shaping these mechanisms in Chinese kindergarten teachers.

## 1 Introduction

Professional capability enhancement of teachers is a pivotal focus in this era of high-quality educational development. As frontline practitioners in preschool education, kindergarten teachers’ expertise directly determines the quality of early childhood education and is a cornerstone of its advancement. Teachers’ professional identity is a complex of their positive cognition, experiences, and behavioral tendencies toward the profession and internalized professional roles (Wei et al., 2013). It represents a teacher’s positive attitude toward the profession and is a part of positive occupational psychology research. Low professional identity in kindergarten teachers can have various negative effects. For example, weak professional identity is closely associated with high job burnout and turnover intention (Ding and Xie, 2021; Lin, 2019), thereby undermining the stability of the teaching workforce. Consequently, investigating the mechanisms that influence kindergarten teachers’ professional identities is crucial for enhancing their work quality and promoting children’s holistic development.

Perceived organizational support refers to employees’ beliefs about the extent to which an organization values their contributions and cares about their well-being (Eisenberger et al., 1986). The conservation of resources theory (Hobfoll, 2011) posits that individuals strive to obtain, retain, and protect internal psychological and external resources essential for maintaining their position and facilitating growth. The dynamics of resource gain and loss significantly impact an individual’s overall psychological state (Hobfoll, 2011). Organizational support is a vital external resource for kindergarten teachers, helping them replenish psychological, personal, and conditional resources, thereby fostering the formation and consolidation of job satisfaction (Chen, 2025; Oubibi et al., 2022). Previous research has identified perceived organizational support as a key influencer of teachers’ professional identities (Yao et al., 2023). Adequate organizational support encourages teachers to invest more in their work, strengthening their professional identities. Therefore, this study aimed to provide a reference for enhancing kindergarten teachers’ professional identity from the perspective of perceived organizational support, offering targeted and feasible suggestions for improving the quality and stability of the early childhood teaching workforce.

### 1.1 Teachers’ achievement goal orientation as a mediator

Schools are achievement arenas for both students and teachers, who pursue success in their roles but differ in their definition of success, goals they prioritize, and achievement goal orientations (Butler, 2007). Butler (2007) proposes a four-dimensional framework of teachers’ achievement goal orientations: mastery (learning goal orientation), ability approach (performance approach goal orientation), ability avoidance (performance avoidance goal orientation), and work avoidance (work avoidance goal orientation). Specifically, learning goal orientation refers to teachers’ engagement in their work to acquire and develop professional knowledge and skills; performance approach, strive to demonstrate excellent teaching abilities; performance avoidance, avoidance of displaying lower teaching competence; and work avoidance, tendency to minimize work engagement and exert minimal effort in daily work. In other words, these goal orientations reflect teachers’ motivational tendencies in an instructional context.

Achievement goal theory (Dweck, 1986; Ames, 1992) highlights that achievement goals result from the interplay of environmental factors (e.g., organizational motivational climate), learning experiences, and learner characteristics, which shape individuals’ evaluations and perceptions of their job and work style. Teachers’ achievement goal orientation may serve as a crucial internal mechanism linking perceived organizational support and professional identity. School-level factors, including a positive feedback culture and autonomy-supportive leadership, are positively associated with teachers’ learning goal orientation (Dickhäuser et al., 2021). An international study of university instructors from Germany, the USA, and India revealed the positive association of learning goal orientation and negative association of work avoidance orientation with positive affect, teaching quality, and professional learning (Daumiller et al., 2022). Furthermore, a mutually reinforcing relationship was identified between teachers’ performance approach goal orientation and job satisfaction, indicating that the pursuit of performance-oriented goals positively influences job satisfaction, enhancing their adoption (Rinas et al., 2023).

Research on the mediating role of teachers’ achievement goal orientation in the relationship between perceived organizational support and professional identity is limited. Nonetheless, a relevant study by Luo et al. (2020) found that teachers’ mastery goals mediate the relationship between transformational leadership and job satisfaction. This indirectly supports the potential mediating role of the proposed model. Therefore, this study aimed to explore how perceived organizational support influences kindergarten teachers’ achievement goal orientation and, in turn, affects their professional identity.

### 1.2 Job stress as a moderator

Job stress is defined as the psychological and physiological strain experienced by educators owing to factors such as workload, work intensity, and role conflict (Kyriacou, 2001). Early childhood educators operate in a multifaceted environment, confronting challenges, including individual differences between children, parental expectations, and demanding instructional responsibilities, all of which contribute to heightened stress levels (Lu et al., 2019). Subjective assessment of stress guides goal selection. The challenge and hindrance framework indicates that different types of stress have distinct effects on motivation. Challenge stress is more likely to support learning and mastery orientations, whereas hindrance stress, performance and avoidance orientations (Horan et al., 2020; Kubicek et al., 2023). Empirical evidence suggests that different types of job stress can cause tension and erode performance. However, challenge stress may be accompanied by more positive task engagement under certain conditions, indicating a nuanced dynamic relationship between job stress and achievement goal orientation (Pindek et al., 2024). Recent systematic reviews and empirical studies have shown that teachers’ job stress is negatively associated with engagement and efficacy and that such motivational states typically co-occur with lower achievement goal orientation and greater avoidance orientation (Daniel and Van Bergen, 2023; Han and Gao, 2023).

According to the job demands-resources model (Bakker and Demerouti, 2007), work imposes physical, psychological, social, and organizational demands on individuals, leading to fatigue and tension. However, job resources help them cope effectively, thereby promoting their engagement and well-being (Luo et al., 2025). Organizational support is an important work resource that can transform school-provided training, feedback, and emotional support into a positive motivational orientation and engagement. In contrast, higher environmental stress reduces the efficiency of the transformation toward adaptive goals, reducing the positive effect of perceived organizational support on goal orientation; this positive effect completely manifests when stress is lower (Bakker and Demerouti, 2007, 2017).

Recent empirical studies have supported this regulatory perspective. For example, in healthcare and education, perceived organizational support changes the direction and intensity of the impact of work requirements on outcome variables: high perceived organizational support more effectively weakens negative consequences when the requirements are higher, consistent with the buffering proposition of the model (Ramaci et al., 2024). In a sample of Chinese teachers, perceived organizational support was positively and stably correlated with engagement and mental health, suggesting a systematic connection between workload and support and engagement, in line with the path assumption of the work requirements resource model (Wang, 2024). Another large-scale study showed that perceived organizational support interacted with stress to influence profession-related psychological constructs in teachers. Moreover, higher levels of perceived organizational support reduced the negative impact of stress on professional identity, providing group-based empirical evidence of the boundary condition in which high stress weakens resource transformation, whereas low stress strengthens it (Liang et al., 2022).

### 1.3 The present study

**Figure 1:**
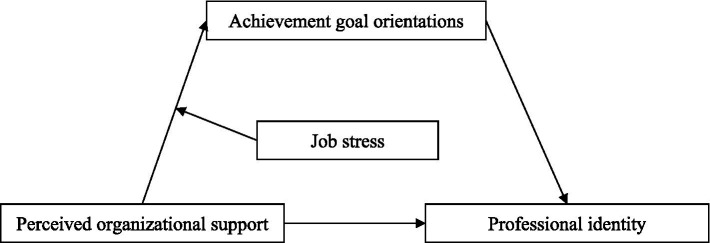
The hypothesized model.

We examined a moderated mediation model (Figure 1) with the following hypotheses:

*H1*: Teachers’ achievement goal orientations significantly mediate the relationship between perceived organizational support and professional identity.

*H2*: Job stress significantly moderates the relationship between perceived organizational support and achievement goal orientations.

## 2 Materials and methods

### 2.1 Participants

This study randomly selected multiple kindergartens across the country willing to participate in the research and invited all teachers from these kindergartens to complete a questionnaire survey. A total of 1,627 teachers participated in the survey. Subsequently, 152 questionnaires were excluded due to incomplete answers, completion times of less than 180 s and abnormal age filling. Finally, 1,475 kindergarten teachers (97% female, 3% male) were included in the analysis. The participants’ ages ranged from 20 to 60 years (*M_age_* = 34.01 years, *SD* = 8.41 years). In terms of their teaching experience, participants’ years of experience ranged from 0 to 43 years (*M* = 10.92 years, *SD* = 9.33 years). The majority of participants worked in Guangdong (62.2%), Shandong (23.7%), and other provinces, with the remainder distributed across other regions. Regarding the participants’ academic and professional details, most teachers were employed in public schools (85.3%), while a smaller proportion worked in private schools (14.7%). In terms of employment status, the majority were full-time employees (85.5%), with a small percentage in temporary or contracted positions.

### 2.2 Procedure

For convenience, data were collected using the Wenjuanxing platform.^1^ Ethical approval was obtained from the Academic Ethics Review Board at the researchers’ university before the survey. Questionnaire links were distributed to teachers only after securing their informed consent. All respondents were asked to provide basic demographic information and complete questionnaires assessing their perceived organizational support, professional identity, achievement goal orientations, and job stress.

### 2.3 Measures

#### 2.3.1 Perceived organizational support

The Chinese version of the Teacher Perceived Organizational Support Scale developed by Ling et al. (2006) was adopted for this research to assess perceived organizational support among teachers. This scale includes 12 items (e.g., “When I encounter problems in my work, I will get the help from the school”). Answers to the items are given on a five-point scale ranging from 1 (“completely disagree”) to 5 (“completely agree”). A higher score indicated a higher degree of perceived organizational support among teachers. The scales in this study demonstrated strong reliability and validity, with Cronbach’s *α* = 0.97, composite reliability (CR) = 0.97, and average variance extracted (AVE) = 0.76.

#### 2.3.2 Job stress

The Chinese version of Teacher’s Job Stress Scale (Tang, 2018) was used to measure the teacher’s Job Stress, which consists of 5 items (e.g., “All kinds of evaluation, inspection often troubled me, so that I can’t lift the spirit”). Answers to the items are given on a five-point scale ranging from 1 (“completely disagree”) to 5 (“completely agree”). A higher score indicated a higher degree of job stress among teachers. The scales in this study demonstrated strong reliability and validity, with Cronbach’s α = 0.94, CR = 0.95, and AVE = 0.80.

#### 2.3.3 Teachers’ achievement goal orientations

Teachers’ achievement goal orientations were measured by a brief scale for teachers’ achievement goal orientations (Janke et al., 2019). This scale includes 12 items and encompasses four dimensions: learning goal orientation (e.g., “I aspire to improve my pedagogical knowledge and competence”), performance approach goal orientation (e.g., “I aspire to demonstrate that I know more than other teachers”), performance avoidance goal orientation (e.g., “I aspire to conceal when I do something less satisfying than other teachers”) and work avoidance goal orientation (e.g., “I aspire not to have to work too hard”). In this study, the scale was translated into Chinese by the researchers following a standard translation-back-translation procedure. The translated version was pilot-tested with a small sample of teachers to assess clarity and cultural relevance. Answers to the items are given on a seven-point scale ranging from 1 (“completely disagree”) to 7 (“completely agree”). A higher score indicated tendency toward this particular achievement goal orientation among teachers. The four scales demonstrated excellent reliability and convergent validity, with Cronbach’s *α* coefficients of 0.99, 0.94, 0.90, and 0.92; CR values of 0.99, 0.96, 0.94, and 0.95; and AVE values of 0.97, 0.90, 0.84, and 0.87, respectively.

#### 2.3.4 Professional identity

Professional identity was assessed by a Chinese version of Teacher Professional Identity Scale (Li and Yan, 2018). This scale includes 9 items (e.g., “I usually feel confident when dealing with teacher work”). Answers to the items are given on a five-point scale ranging from 1 (“completely disagree”) to 5 (“completely agree”). A higher score indicated a higher degree of professional identity among teachers. The scales in this study demonstrated strong reliability and validity, with Cronbach’s α = 0.95, CR = 0.96, and AVE = 0.75, respectively.

### 2.4 Data analyses

Prior to hypothesis testing, the normality of the data was examined by assessing skewness and kurtosis. All variables were found to be within acceptable ranges (skewness < |2.0| and kurtosis < |7.0|). Furthermore, all independent variables had variance inflation factors below 3, suggesting no multicollinearity concerns. To assess common method bias, Harman’s single-factor test was conducted. The analysis extracted six factors with eigenvalues exceeding 1, with the largest factor explaining 36.74% of the variance, which is below the 40% threshold. This indicates that common method bias was not a significant issue in this study.

Using SPSS 27.0, the study first performed descriptive statistics and bivariate correlations for the research variables. Second, to examine the mediating role of kindergarten teachers’ four types of achievement goal orientations and the moderating role of job stress, two structural equation models were constructed using Mplus 8.0 (Muthén and Muthén, 2017). The comparative fit index (CFI; acceptable > 0.90), the root mean square error of approximation (RMSEA; acceptable < 0.08) with its 90% confidence interval (CI), the standardized root mean square residual (SRMR; acceptable < 0.08), and a nonsignificant chi-square statistic (*χ^2^*) were employed to assess the overall model fit. If the interaction effect between perceived organizational support and job stress on achievement goal orientations was significant, we further probed the simple slopes and the conditional mediation effects between participants with higher levels of job stress (*M* + SD) and those with lower levels of job stress (*M* − SD). To assess the significance of (conditional) mediation effects, we used bootstrapping (*n* = 1,000) and its 95% CI. The (moderated) mediation effect would be considered tenable if 0 was excluded from the 95% CI.

## 3 Results

### 3.1 Descriptive statistics and correlations

Table 1 displays the descriptive statistics of the major research variables and their bivariate correlations. Specifically, perceived organizational support was positively associated with four types of achievement goal orientations and professional identity, but negatively associated with job stress. Job stress was positively associated with four types of achievement goal orientations and professional identity, too. Besides, four types of achievement goal orientations were positively associated with professional identity.

**Table 1:** Correlations and descriptive statistics of the variables.

Variable	1	2	3	4	5	6	7	8	9
1. POS	1.00								
2. JS	−0.14***	1.00							
3. Learning GO	0.41***	0.08**	1.00						
4. Performance approach GO	0.27***	0.32***	0.38***	1.00					
5. Performance avoidance GO	0.19***	0.46***	0.20***	0.72**	1.00				
6. Work avoidance GO	0.05*	0.53***	0.20***	0.48***	0.62***	1.00			
7. PI	0.49***	0.06*	0.62***	0.35***	0.21***	0.21***	1.00		
8. Age	0.02	0.03	0.06*	0.05	0.01	−0.02	0.17***	1.00	
9. TE	0.02	0.11***	0.06*	0.09**	0.08**	0.05	0.17***	0.85***	1.00
*M*	4.09	3.30	6.34	5.31	4.75	5.03	4.43	34.01	10.92
*SD*	0.82	1.12	0.95	1.52	1.67	1.63	0.64	8.41	9.33

POS, perceived organizational support; JS, job stress; Learning GO, learning goal orientation; Performance approach GO, performance approach goal orientation; Performance avoidance GO, performance avoidance goal orientation; Work avoidance GO, work avoidance goal orientation; PI, professional identity; TE, teaching experience. *p < 0.05, **p < 0.01, ***p < 0.001.

### 3.2 Testing for mediation effect

**Figure 2:**
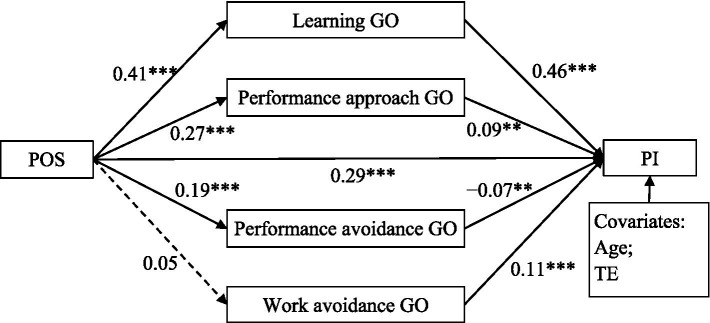
The mediation model. POS, perceived organizational support; Learning GO, learning goal orientation; Performance approach GO, performance approach goal orientation; Performance avoidance GO, performance avoidance goal orientation; Work avoidance GO, work avoidance goal orientation; PI, professional identity; TE, teaching experience. **p < 0.01, ***p < 0.001.

The mediation model exhibits good fit [*χ^2^* = 37.59, *df* = 10, RMSEA = 0.04 (90% CI = (0.03, 0.06), CFI = 0.99, TLI = 0.98, SRMR = 0.03)]. This model explained 48.30% variance in professional identity. As shown in Figure 2, after controlling for age and teaching experience, perceived organizational support was positively associated with learning goal orientation, performance approach goal orientation and performance avoidance goal orientation, but not with work avoidance goal orientation. In addition, perceived organizational support, learning goal orientation, performance approach goal orientation and work avoidance goal orientation were positively associated with professional identity, whereas performance avoidance goal orientation was negatively associated with professional identity. Further mediation analyses are presented in Table 2. The results indicated that the mediation effect of work avoidance goal orientation was not significant, but the mediation effect of learning goal orientation, performance approach goal orientation and performance avoidance goal orientation was significant.

**Table 2:** Summary of the direct and indirect effects.

Specific pathways tested in the model	Effect	*SE*	95% CI
Direct pathway			
POS → PI	0.29	0.03	[0.24, 0.35]
Indirect pathways			
POS → Learning GO → PI	0.18	0.02	[0.14, 0.22]
POS → Performance approach GO → PI	0.02	0.01	[0.01, 0.04]
POS → Performance avoidance GO → PI	−0.01	0.01	[−0.03, −0.01]
POS → Work avoidance GO → PI	0.01	0.00	[−0.00, 0.01]

POS, perceived organizational support; Learning GO, learning goal orientation; Performance approach GO, performance approach goal orientation; Performance avoidance GO, performance avoidance goal orientation; Work avoidance GO, work avoidance goal orientation; PI, professional identity.

### 3.3 Testing for moderated mediation

**Figure 3:**
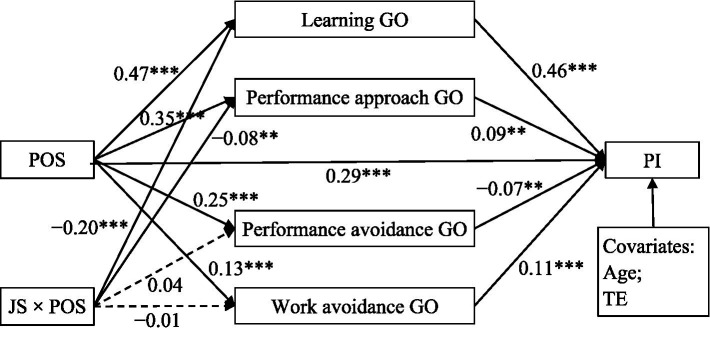
The moderated mediation model. POS, perceived organizational support; JS, job stress; Learning GO, learning goal orientation; Performance approach GO, performance approach goal orientation; Performance avoidance GO, performance avoidance goal orientation; Work avoidance GO, work avoidance goal orientation; PI, professional identity; TE, teaching experience. **p < 0.01, ***p < 0.001.

Building on the aforementioned mediation model, this study further examined whether job stress would moderate the relationships between perceived organizational support and each of the four achievement goal orientations. The moderated mediation model depicted in Figure 3 exhibits good fit [*χ^2^* = 37.25, *df* = 10, RMSEA = 0.04 (90% CI = (0.03, 0.06), CFI = 0.99, TLI = 0.98, SRMR = 0.02)]. This model explained 48.50% variance in professional identity. The results indicated that job stress did not moderate the association between perceived organizational support and performance avoidance goal orientation as well as work avoidance goal orientation, but it served as a significant moderator in the association between perceived organizational support and learning goal orientation as well as performance approach goal orientation.

**Figure 4:**
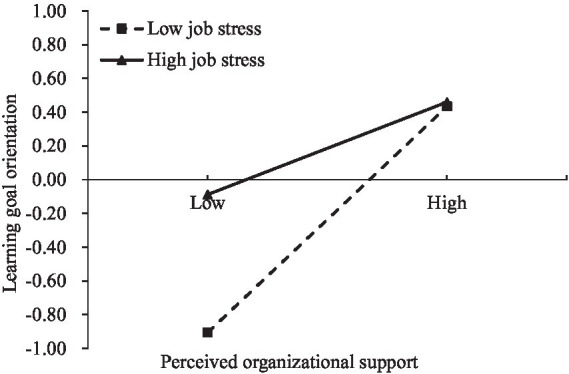
The moderating effect of job stress on the relationship between perceived organizational support and learning goal orientation.

**Figure 5:**
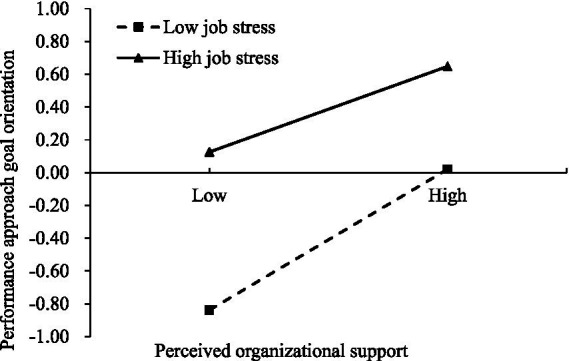
The moderating effect of job stress on the relationship between perceived organizational support and performance approach goal orientation.

To better understand the moderating role of job stress, a follow-up simple slope analysis was conducted. As shown in Figure 4, the results indicated that, regardless of the level of perceived organizational support, participants with high job stress exhibited higher learning goal orientation compared to those with low job stress. For teachers possessing low job stress, the association between perceived organizational support and learning goal orientation was stronger (*β*_simple_ = 0.66, *SE* = 0.05, *t* = 12.28, *p* < 0.001) than it was for teachers possessing high job stress (*β*_simple_ = 0.29, *SE* = 0.03, *t* = 9.78, *p* < 0.001). As shown in Figure 5, the results indicated that, regardless of the level of perceived organizational support, participants with high job stress also exhibited higher performance-approach goal orientation compared to those with low job stress. For teachers possessing low job stress, the association between perceived organizational support and performance-approach goal orientation was stronger (*β*_simple_ = 0.42, *SE* = 0.03, *t* = 9.53, *p* < 0.001) than it was for teachers possessing high job stress (*β*_simple_ = 0.27, *SE* = 0.04, *t* = 9.58, *p* < 0.001).

We further examined whether the mediating effect of perceived organizational support on professional identity via learning goal orientation as well as performance approach goal orientation was conditioned by job stress. The bias-corrected percentile bootstrap results (see Table 3) showed that the mediating effects of learning goal orientation in the pathway from perceived organizational support to professional identity was stronger in teachers with low job stress than in those with high job stress. In addition, the mediating effects of performance approach goal orientation in the pathway from perceived organizational support to professional identity was also stronger in teachers with low job stress than in those with high job stress. Specifically, the impact of high job stress diminishes as perceived organizational support increases.

**Table 3:** Conditional indirect effects of perceived organizational support on professional identity via learning goal orientation and performance approach goal orientations by levels of job stress.

Levels of job stress	Indirect effect	*SE*	95% CI
Learning GO as mediator			
Low (*M* − SD)	0.30	0.04	[0.22, 0.36]
Med (*M*)	0.21	0.02	[0.17, 0.27]
High (*M* + SD)	0.13	0.022	[0.10, 0.17]
Performance approach GO as mediator			
Low (*M* − SD)	0.04	0.01	[0.02, 0.06]
Med (*M*)	0.03	0.01	[0.01, 0.05]
High (*M* + SD)	0.02	0.01	[0.01, 0.04]

Learning GO, learning goal orientation; Performance approach GO, performance approach goal orientation.

## 4 Discussion

### 4.1 The direct effect of perceived organizational support on professional identity

This study reaffirms the direct effect of perceived organizational support on kindergarten teachers’ professional identities (Yao et al., 2023). This finding underscores the fact that perceived institutional support, such as tangible resources, supervisory fairness, and collegial respect, are positively associated with teachers’ sense of competence, meaning, and belonging in their profession. This finding is consistent with the conservation of resources theory (Hobfoll, 2011), which posits that supportive work environments are key resources that help employees achieve their goals and foster positive work-related attitudes and stronger identification with their professional roles.

### 4.2 The mediating effect of teachers’ achievement goal orientations

Three types of achievement goal orientations mediated the relationship between perceived organizational support and professional identity, partly supporting H1. In particular, achievement goal dimensions played different mediating roles in the relationship between perceived organizational support and professional identity. Of these, the largest effect size was observed in the mediating pathway of learning goal orientation, highlighting its significant positive impact on kindergarten teachers.

The results showed that perceived organizational support can enhance teachers’ professional identity by positively influencing their learning and performance approach goal orientations. In other words, perceived organizational support can enhance teachers’ professional identities by encouraging them to improve and demonstrate their abilities. This finding supports the achievement goal theory (Dweck, 1986; Ames, 1992), which posits that an organizational motivational climate enhances employees’ expectations of rewards for their efforts. Organizational support may provide opportunities for teachers to showcase their abilities and, through positive feedback and recognition, reinforce their professional identities (Dickhäuser et al., 2021; Daumiller et al., 2022). This finding aligns with that observed by Qin and Liu (2023) that principal support has a significant positive influence on teachers’ professional learning and drives individuals to pursue success.

In contrast, perceived organizational support positively influenced performance avoidance goal orientation, exerting a negative impact on professional identity. Research indicates that teachers with a strong performance avoidance goal orientation are more likely to experience stress, burnout, and turnover intention, which, in turn, weaken their sense of professional identity (Li et al., 2021). Focus on avoiding failure, rather than striving for success, may influence teachers in withdrawing from professional development. This avoidance behavior diminishes their ability to benefit from the perceived organizational support, making it more difficult to enhance their teaching skills or achieve professional accomplishment.

Notably, work avoidance goal orientation did not significantly mediate the relationship between perceived organizational support and professional identity. Specifically, perceived organizational support did not significantly influence work avoidance goal orientation; however, work avoidance goal orientation significantly positively impacted professional identity. In other words, even high levels of organizational support may not change teachers’ work avoidance goal orientations. Furthermore, although work avoidance goal orientation is often regarded as a negative motivation, its meaning may differ in teachers’ real working environments. Given their high-intensity workload, even highly dedicated educators may occasionally seek brief respites to more effectively allocate their time and resources (Bakker and De Vries, 2021; Butler, 2007). For instance, kindergarten teachers may reduce lesson preparation time to manage administrative duties or utilize free periods for professional development activities. Therefore, work avoidance should not be equated with mere disengagement or indicate a lack of professional identity. In certain situations, moderate work avoidance may reflect a strategic time management approach rather than an evasion of professional responsibilities. Future research should explore the specific mechanisms through which work avoidance operates in different teaching contexts.

### 4.3 The moderating effect of job stress

Job stress significantly moderated the relationship between perceived organizational support and the two types of adaptive goal orientations, partially supporting H2. The positive correlation between perceived organizational support and learning and performance approach goal orientations was stronger in low stress but weaker at high stress levels. Additionally, the high-stress group had higher baseline levels of learning and performance approach goal orientations regardless of the support level. This is consistent with the work requirements-resource model and resource conservation theory: low stress implies more abundant available attention and recovery resources, transforming external support into learning and performance approach goal orientations, while high stress implies resource occupation and threat assessment, reducing the transformation possibility of the same support level (Bakker and Demerouti, 2017; Hobfoll et al., 2018). Regarding the finding of higher baseline levels in the high-stress group, the distinction between challenges and obstacles provides supplementary explanations: when stress is evaluated as a challenge, cognitive and growth-oriented requirements positively correlate with learning and current performance, elevating overall learning or performance approach goal tendencies; however, it does not negate the weakening of the slope of this relationship under high-stress conditions (Kubicek et al., 2023; Pindek et al., 2024). This aligns with previous conclusions that adaptiveness goals are linked to positive practices, suggesting that this connection is regulated by stress levels (Han and Gao, 2023).

Job stress significantly moderated the mediating role of achievement goal orientation in the relationship between perceived organizational support and professional identity. Specifically, the indirect effect of perceived organizational support on professional identity through learning and performance approach goal orientations was stronger under low-stress and weaker under high-stress conditions. This is consistent with the theoretical path of “resources acting through motivational channels on key attitude outcomes”; stress means the loss of resources and competition, reducing the efficiency of the channel (Bakker and Demerouti, 2007; Hobfoll et al., 2018). Furthermore, this aligns with previous research results that increased stress weakens the impact of the same support level on key outcomes (Zhang et al., 2024). This also supports the classic finding that the buffering effect of support is limited by stress level (Wallace et al., 2009), providing a basis for designing differentiated support schemes based on stress levels.

However, job stress did not significantly moderate the relationship between perceived organizational support and performance- and work avoidance goal orientations, partially contradicting H2. This may be related to the stability and defensiveness of avoidance orientation. A recent meta-analysis has shown that performance avoidance goal orientation has a moderate positive correlation and learning goal orientation has a weak negative correlation with internalizing symptoms such as anxiety and depression. This suggests that avoidance has a lower immediate sensitivity to general external support (Diaconu-Gherasim et al., 2024). The latest evidence on work avoidance goal orientation also shows that it often co-occurs with low control and energy-saving strategies and remains relatively stable in the context of negative emotions, which is difficult to be changed by short-term support (Ebert et al., 2024).

### 4.4 Implications and limitations

This study examined the potential impact mechanism of perceived organizational support on kindergarten teachers’ professional identity, providing a novel perspective of understanding professional identity. This study has significant theoretical and practical implications. First, it confirmed the crucial role of perceived organizational support. This finding emphasizes the necessity to prioritize improving the external environment, resources, and organizational support in all aspects for preschool teachers, thereby strengthening their professional identity. Second, the study revealed the mediating roles of three key achievement goals in the relationship between teachers’ perceived organizational support and professional identities. Learning and performance approach goal orientations are the core driving forces behind professional identity, while performance avoidance goal orientation is not conducive to it. Therefore, kindergartens should focus on guiding and supporting teachers to form positive achievement goal orientations, thereby improving their sense of professional identity. Finally, although high job stress may contribute to positive achievement goal orientation, excessive stress can weaken the benefits of perceived organizational support and be detrimental to teachers’ professional identity and growth. Therefore, along with fostering healthy goals, schools must remain attentive to teachers’ job stress.

However, this study has certain limitations. First, its cross-sectional design made it challenging to establish definitive causal relationships. Future research should employ longitudinal designs to further validate these findings. Second, reliance on teachers’ self-reports as the sole data source may introduce subjective biases, potentially compromising the objectivity of the results. To minimize potential social desirability effects, future studies should incorporate objective indicators, such as organizational support measures from kindergarten administrative records and teachers’ professional performance assessment by colleagues or parents. Finally, this study focused primarily on teachers’ perceived organizational support and internal factors, such as achievement goal orientation and job stress. However, external factors such as family support, community environment, and national educational policies could also influence professional identity. Future research should consider incorporating these external variables to provide a more holistic understanding of the factors shaping teachers’ professional identities.

## 5 Conclusion

This study investigated the differences in the potential mechanisms by which perceived organizational support affects professional identity in kindergarten teachers in China. The results indicated significant mediating effects of learning, performance approach, and performance avoidance goal orientations. Moreover, job stress played an important moderating role in the correlation between perceived organizational support and learning and performance approach goal orientations. Specifically, regardless of the perceived organizational support level, teachers with high job stress exhibited higher learning and performance approach goal orientations than those with low job stress. The mediating role of achievement goal orientation in the relationship between perceived organizational support and professional identity was more pronounced in teachers who experienced low levels of job stress. This study provides a robust theoretical foundation and practical guidance for developing strategies to improve kindergarten teachers’ professional identities.

## Data Availability

The raw data supporting the conclusions of this article will be made available by the authors, without undue reservation.

## Footnotes

1
www.wjx.cn
